# A Predictive Tool Based on DNA Methylation Data for Personalized Weight Loss through Different Dietary Strategies: A Pilot Study

**DOI:** 10.3390/nu15245023

**Published:** 2023-12-06

**Authors:** Nereyda Carolina García-Álvarez, José Ignacio Riezu-Boj, J. Alfredo Martínez, Sonia García-Calzón, Fermín I. Milagro

**Affiliations:** 1 Center for Nutrition Research, Department of Nutrition, Food Science and Physiology, Faculty of Pharmacy and Nutrition, University of Navarra, 31008 Pamplona, Spain; ngarciaalva@alumni.unav.es (N.C.G.-Á.); jiriezu@unav.es (J.I.R.-B.); jalfmtz@unav.es (J.A.M.); sgcalzon@unav.es (S.G.-C.); 2 Navarra Institute for Health Research (IdiSNA), 31008 Pamplona, Spain; 3 Centro de Investigación Biomédica en Red de la Fisiopatología de la Obesidad y la Nutrición (CIBERobn), Carlos III Health Institute, 28029 Madrid, Spain

**Keywords:** BMI loss, epigenetic score, precision nutrition, hypocaloric diet, predictive model

## Abstract

Background and aims: Obesity is a public health problem. The usual treatment is a reduction in calorie intake and an increase in energy expenditure, but not all individuals respond equally to these treatments. Epigenetics could be a factor that contributes to this heterogeneity. The aim of this research was to determine the association between DNA methylation at baseline and the percentage of BMI loss (%BMIL) after two dietary interventions, in order to design a prediction model to evaluate %BMIL based on methylation data. Methods and Results: Spanish participants with overweight or obesity (*n* = 306) were randomly assigned to two lifestyle interventions with hypocaloric diets: one moderately high in protein (MHP) and the other low in fat (LF) for 4 months (Obekit study; ClinicalTrials.gov ID: NCT02737267). Basal DNA methylation was analyzed in white blood cells using the Infinium MethylationEPIC array. After identifying those methylation sites associated with %BMIL (*p* < 0.05 and SD > 0.1), two weighted methylation sub-scores were constructed for each diet: 15 CpGs were used for the MHP diet and 11 CpGs for the LF diet. Afterwards, a total methylation score was made by subtracting the previous sub-scores. These data were used to design a prediction model for %BMIL through a linear mixed effect model with the interaction between diet and total score. Conclusion: Overall, DNA methylation predicts the %BMIL of two 4-month hypocaloric diets and was able to determine which type of diet is the most appropriate for each individual. The results of this pioneer study confirm that epigenetic biomarkers may be further used for precision nutrition and the design of personalized dietary strategies against obesity.

## 1. Introduction

Obesity is considered one of the main factors of morbidity due to malnutrition, and it is observed how incidences increase over the years. Obesity and overweight are defined as an excessive or abnormal accumulation of fat that can be detrimental to health. It is classified by body mass index (BMI). It is determined as overweight when presenting a BMI of 25 to 29.9 kg/m^2^, and obesity is defined as when a person has a BMI of 30 kg/m^2^ or more [1]. 

The pathogenesis of obesity is due to a metabolic condition of disturbed adipocyte function and low-grade systemic inflammation, and this can induce epigenetic changes that perpetuate inflammation [2]. Among the most studied epigenetic mechanisms is DNA methylation (mDNA) [3], which is considered to be a key part of the pathogenesis and clinical manifestations of obesity [4]. Methylation marks are chemical modifications of the structure and function of DNA, in which there is no variation in the genetic code but, rather, in the expression of the genes. Epigenetic mechanisms make the DNA code available or unavailable for translation into gene products [5]. DNA methylation occurs when a methyl group is added to the 5′ carbon at the cytosine base-binding sites on the CpG dinucleotides of DNA. In general, in the genome of a differentiated cell, there may be regions that are both methylated and unmethylated [6]. It has previously been shown that methylation can influence the development of obesity but also the response to dietary weight loss treatments [7,8]. This is due to the different complex metabolic mechanisms in which methylation may be involved [9]. It has been observed that methylation modulates molecular mechanisms associated with adipogenesis [10,11]. It has also been demonstrated that methylation in genes related to hunger and satiety mechanisms contribute to the development of obesity [12], and the same happens when methylation is present in genes that have a function in the thermogenesis of adipose tissue that regulates energy expenditure [13]. It has been shown that epigenetic changes can be a transgenerational inheritance [14]; however, these epigenetic changes are modifiable in response to lifestyle and environment [15], as has been shown when studying the interaction of epigenetics and diet [16,17]. Therefore, the integration of epigenetics is considered relevant for the management and prevention of obesity [18]. The use of precision nutrition is proposed, since it contemplates metabolic phenotyping through high-performance omics technologies such as epigenomics [19] and takes into account lifestyle factors such as exercise, alcohol consumption, and general nutritional and metabolic status [20]. For this reason, it has been seen that, if the nutritional plans are adapted according to the characteristics and needs of each individual or in groups of people who have similar characteristics, there are more satisfactory results for treating chronic diseases [21]. Within this perspective, the aim of this research was to determine the association between basal DNA methylation and the percentage of BMI loss after a dietary intervention, in order to design a model that, based on a methylation score, predicts the percentage of BMI loss with two different types of diets.

## 2. Materials and Methods

### 2.1. Study Population

The population selected in this research was from the Obekit study, in which 314 Spanish individuals with overweight and obesity were initially recruited. The study lasted from October 2015 to February 2017 and was carried out in the Metabolic Unit of the Nutrition Research Center of the University of Navarra.

**Figure 1:**
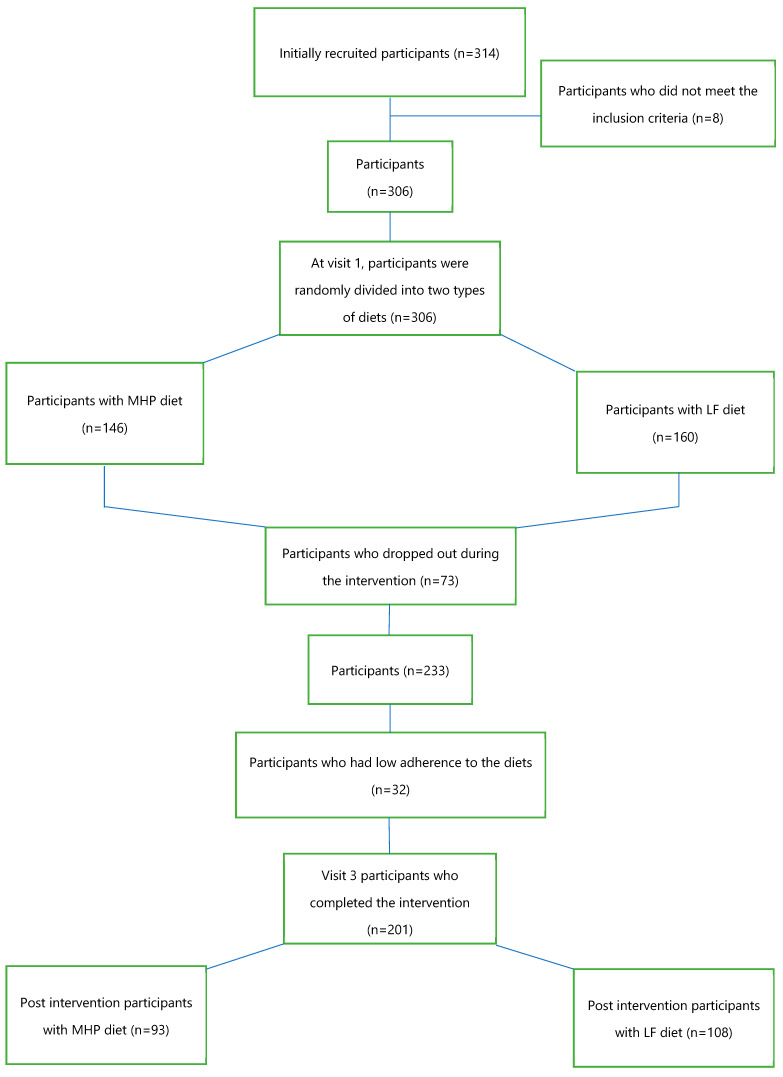
Flow chart of the participants who started and completed the intervention.

The inclusion criteria were participants with an age range of 18–67 years old and participants with overweight (BMI: 25–29.9 kg/m^2^) or with obesity (BMI: 30–40 kg/m^2^). Major exclusion criteria were type 1 diabetes mellitus, pregnant or breastfeeding women, cardiovascular disease, cancer, and eating and cognitive disorders. Of the 314 subjects initially recruited, 8 did not meet the inclusion criteria. The intervention began with 306 participants, who were randomly assigned to two types of hypocaloric diets: 146 participants with a moderately high-protein diet (MHP) and 160 participants with a low-fat diet (LF). The intervention lasted 4 months (Figure 1). Finally, 233 participants completed the dietary intervention, but only 201 were considered to have good adherence to the diets.

All research procedures were carried out following the ethical principles of the Declaration of Helsinki of 2013 [22]. The study protocol was approved by the Research Ethics Committee of the University of Navarra (ref. 132/2015). This trial was registered at clinicaltrials.gov (ID. NCT02737267; https://clinctrals.gov/ct2/show/NCT02737267?term=NCT02737267&cond=obekit&draw=2&rank=1, accessed on 29 March 2017). All participants gave their written informed consent before their inclusion in the study.

### 2.2. Study Design

Using the Obekit study database, the variables of interest for this research were selected from the 306 participants. General data such as sex; date of birth; and pathological history such as dyslipidemia, hypertriglyceridemia, hypercholesterolemia, and arterial hypertension were taken. The biochemical and anthropometric variables of visit 1, considered the baseline visit, and visit 3, which corresponded to the post-intervention visit, were selected.

### 2.3. Nutritional Intervention

The nutritional intervention lasted 4 months. The diets used for the study had a 30% calorie restriction. The individual energy requirements of each participant were estimated at the beginning, calculating their energy expenditure at rest and during physical activity, to prescribe the hypocaloric diets in a random manner. The macronutrient distribution for the moderately high-protein (MHP) diet was 40% carbohydrate, 30% protein, and 30% fat, and for the low-fat (LF) diet, it was 60% carbohydrate, 18% protein, and 22% fat. Both the LF and MHP diets were designed on the basis of a food exchange system. Participants received detailed information from trained dietitians on portion sizes, dietary patterns/eating schedules, and food preparation techniques.

### 2.4. Anthropometric and Biochemical Determinations

All participants underwent standardized procedures to measure body weight, height, waist circumference, and hip circumference, and body mass index (BMI) was calculated using the formula weight (kg) divided by height in squared meters [23]. Body composition was estimated by bioimpedance (Tanita SC-330, Tanita Corp, Tokyo, Japan) and by DEXA or dual-energy X-ray absorptiometry (Lunar Prodigy, General Electric, Fairfield, MA, USA).

Blood samples were drawn after 12 h of fasting to obtain serum and plasma samples for biochemical determinations at the beginning and at the end of the intervention. Serum glucose, total cholesterol (TC), high-density lipoprotein cholesterol (HDL-C), triglycerides (TG), uric acid, and transaminases were assessed using an automated analyzer (Pentra C200, HORIBA Médica, Kyoto, Japan). Low-density lipoprotein cholesterol (LDL-C) levels were estimated using the Friedelwald formula: total cholesterol − HDL-C − (TGs/5) [24]. Insulin, leptin, adiponectin, C-reactive protein, and TNF-α levels were quantified using commercial ELISA kits (insulin and leptin, Mercodia; Biovendor human adiponectin, ELISA; CRP and TNFα, R&D Systems, Minneapolis, MN, USA). Insulin resistance was estimated using the homeostatic model assessment-insulin resistance index (HOMA-IR) according to the formula fasting insulin (mU/L) × plasma glucose (mmol/L)/22.5. Serum oxidized LDL (oxLDL) levels were measured by a solid-phase two-site competitive ELISA (Mercodia AB, Uppsala, Sweden).

### 2.5. DNA Isolation and Bisulfite Conversion

Blood samples taken at the beginning of the study were centrifuged at 4 °C for 15 min to obtain plasma and isolate the buffy coat fraction. DNA extraction was performed with the “MasterPure” DNA purification kit for blood version II (Epicentre Biotechnologies, Madison, WI, USA), and it was quantified with a spectrophotometer (Nanodrop, Thermo Scientific, Wilmington, DE, USA) and stored at −80 °C. In a second step, 500 ng of DNA was treated with sodium bisulfite using the EZ-96 DNA methylation kit (Zymo Research Corporation, Irvine, CA, USA), to convert unmethylated cytosine residues to uracil, while methylated cytokines remained unchanged.

### 2.6. Array Analysis

The levels of methylated DNA were evaluated using the “Infinium MethylationEPIC BeadChip” kit (Illumina, San Diego, CA, USA), which includes 850,000 methylation sites. Samples were scanned with an “Illumina HiScanSQ” system, and image intensities were extracted with “GenomeStudio v1.9” methylation software (Illumina, CA, USA). The within-array quantile subset normalization (SWAN) method was used to improve the results obtained from the platform, reducing technical variation within and between arrays. The ComBat method was used to adjust for batch effects and remove technical variation. In addition, DNA methylation was corrected for cellular composition (granulocytes, monocytes, B cells, CD8+ cytotoxic cells, CD4+ T-helper cells, and natural killer cells) using Houseman’s algorithm [25].

### 2.7. Design of the BMI Percentage Loss Prediction Model Based on the MHP and LF Diets’ Methylation Data

**Figure 2:**
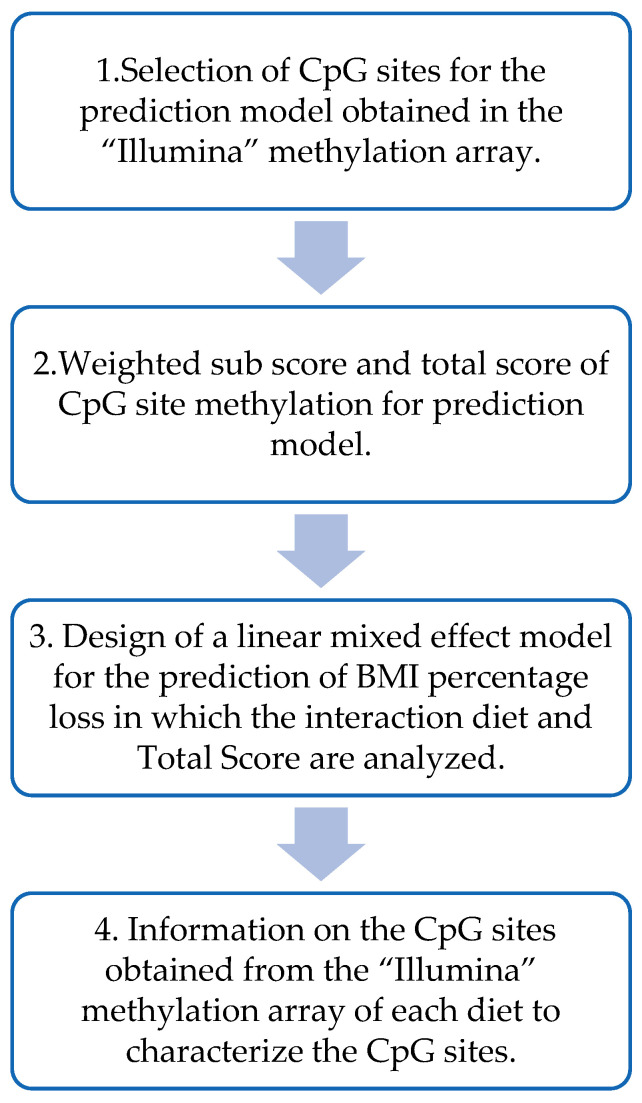
Flow chart for the design of the prediction model based on DNA methylation.

Figure 2 shows the different steps performed to design the prediction model based on DNA methylation data from the beginning of the intervention. Summarizing, the CpGs that presented a significant (*p* < 0.05) Spearman’s correlation with BMI loss for each of the diets were selected. Two weighed sub-scores (one per diet) were built by using the sum of the previously selected CpG sites and multiplying them by the beta coefficients obtained in each of the multiple linear regressions of the MHP diet and the LF diet. To obtain the total score for each individual, the MHP diet sub-score was subtracted from the LF diet sub-score.

Then, a linear mixed effect model was used to predict, based on the total methylation score of each individual, which diet would be the best for the volunteers. This model was designed with the percentage of BMI loss as the dependent variable and the total score, diet (MHP/LF), and the interaction term between the total score and the diet as a fixed effect. And, finally, information from the different CpGs included in the methylation score was obtained from different sources, including Illumina and “UCSC Genome Browser on Human (GRCh37/hg19)”. The biological functions of the genes were searched in the database “GeneCards the human gene database”.

### 2.8. Statistical Analysis

Variables were expressed as the mean ± SEM (standard error of the mean). To characterize the basal anthropometric and biochemical data of the general population, the *p*-value of the comparison of basal means was calculated using the Student’s *t*-test for independent samples between diets MHP and LF. Chi-square was used to calculate the *p*-values for categorical variables such as sex. The differences in the anthropometric and biochemical data of the general population were calculated with the *p*-values of the differences obtained from the baseline and post-intervention data using the Student’s *t*-test for dependent samples for each of the diets. The Student’s *t*-test for independent samples was used to calculate the *p*-value of the comparison of the post-intervention changes of both diets, high-protein (MHP) and low-fat (LF).

### 2.9. Statistical Analysis for the Prediction Model

For the selection of CpG sites obtained in the methylation array, the CpG sites that presented >0.1 standard deviations were chosen. Then, a Spearman’s correlation analysis was performed with the CpG sites correlating their methylation with the percentage of BMI loss and selecting for each diet those that presented *p* < 0.05.

The algorithm “furnival-Wilson leaps and bounds” (vselect in Stata) [26] was used to obtain the best combinations in the multiple linear regression of the percentage of BMI loss with the methylation sites for the MHP diet and LF diet. With the CpG sites obtained by the algorithm, a multiple linear regression was performed to be able to establish the association of the CpG sites with the BMI percentage loss and to be able to use the values of the beta coefficients to construct the weighted methylation sub-scores for each diet and the total methylation score. This total methylation score was used for the design of the BMI percentage loss prediction model, which was performed using a linear mixed effect model. The prediction model was plotted by applying marginals with the diet and the minimum to maximum values of the total methylation score. A Z-test was used to evaluate the distribution of subjects in the prediction model. Statistical analyses were performed with Stata MP 14 (StataCorp LLC, College Station, TX, USA; http://www.stata.com, accessed on 1 February 2023).

## 3. Results

The results show the basal and post-intervention anthropometric and biochemical characteristics of the population with the MHP and LF diet.

### 3.1. Anthropometric and Biochemical Data at Baseline

Statistical analysis of the baseline anthropometric and biochemical data of the 201 participants who were divided into two dietary intervention groups, 93 on the MHP diet and 108 on the LF diet (Figure 1), was performed. Table 1 shows the variables used, the mean, standard error for each diet, and the *p*-value of the comparison of the baseline means. At the beginning, the population did not present statistically significant differences in the anthropometric and biochemical parameters according to the type of diet assigned, except for the circulating levels of TNF-α (*p* = 0.006). The mean values of the baseline variables were similar in both dietary groups. Energy intake did not show significant differences by intervention dietary groups.

### 3.2. Anthropometric and Biochemical Values after the Dietary Intervention and BMI Loss Prediction Model for the MHP and LF Diets Based on DNA Methylation Data

Of the 306 participants, 73 subjects did not complete the dietary intervention, and 32 participants had low adherence to the diets, obtaining post-intervention data from 201 participants: 93 on the MHP diet and 108 on the LF diet (Figure 1).

The statistical analyses of the changes in the anthropometric and biochemical variables in response to dietary treatment after 4 months of intervention are shown in Table 2. All variables showed a significant improvement regardless of the type of diet, demonstrating that the two diets were effective in reducing the anthropometric and biochemical parameters (except for the aspartate aminotransferase (Ast) and TNF-α values, which did not decrease significantly in dietary groups MHP and LF, as well as adiponectin, which did not decrease in the group with the LF diet).

The analysis of the changes after the dietary intervention showed that the HDL-cholesterol in the participants with the LF diet presented a significantly greater increase than in the participants with the MHP diet (*p* = 0.059). Participants with the MHP diet showed a lower decrease in lean mass than participants with the LF diet (*p* = 0.023).

A prediction model based on basal DNA methylation data was designed to determine the percentage of BMI loss for each individual. The two types of diets and the CpG sites with methylation levels at the baseline best associated with BMI reduction were used as predictors.

A selection of methylation sites was made from the 201 participants who completed the dietary intervention and had good adherence to the diets (Figure 1). For this, the mean and standard deviation of the ~850,000 CpG sites of the “Illumina MethylationEPIC” methylation array, which had been adjusted in a previous step for blood cell composition, were calculated. In order to use CpG sites that had sufficient dispersion among the participants, only 1233 CpG sites with a standard deviation >0.1 were used for further analysis.

**Figure 3:**
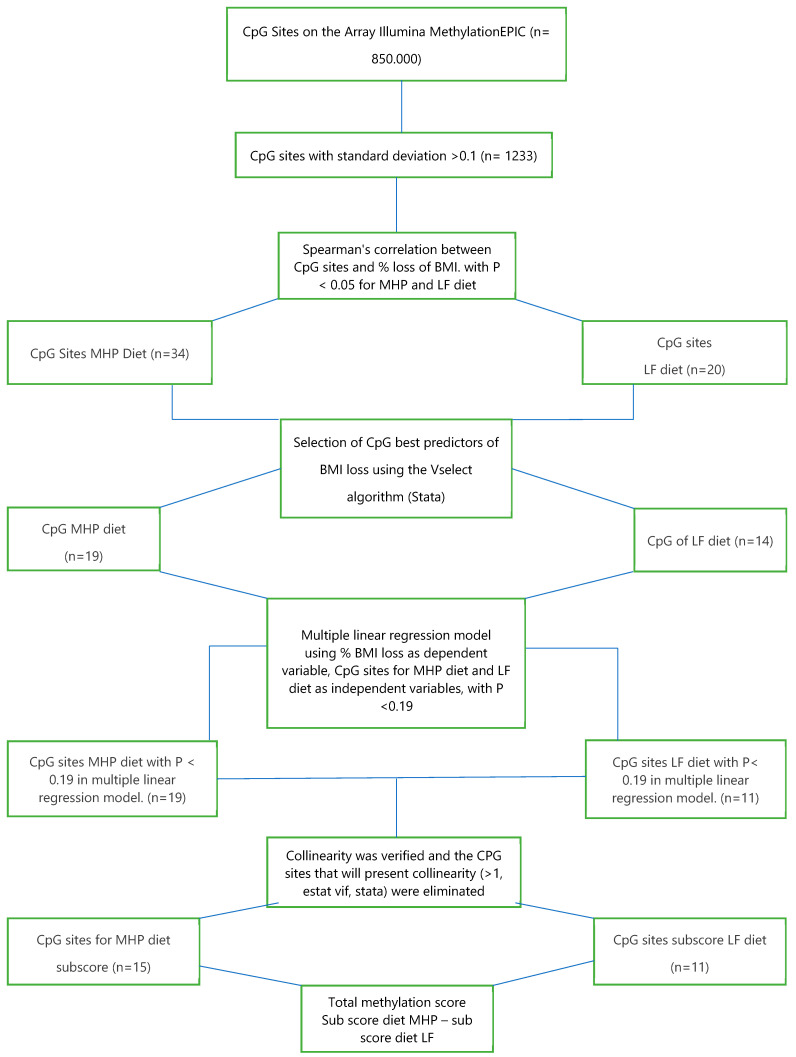
Flow chart for the selection of methylation sites (CpG) used for the prediction model.

Spearman’s correlation was performed between methylation of the selected 1233 CpG sites and the percentage of BMI loss for each of the diets after 4 months of intervention, selecting those CpG sites that presented a significant correlation with *p* < 0.05. Table 3 and Table 4 show the significant CpG sites for each diet (34 for the MHP diet and 20 for the LF diet) (Figure 3). In addition, scatter plots were made between methylation of these significant CpG sites and the change in BMI (Supplementary Figure S1 for the MHP diet and Supplementary Figure S2 for the LF diet).

To better predict the percentage of BMI loss with each of the diets, the “furnival-Wilson leaps and bounds” algorithm (“vselect” in Stata) [26] was used. This algorithm makes it possible to select, among the previously identified CpG sites, those that are best associated with the percentage of BMI loss to be used in a multiple linear regression model. The algorithm recognized 19 out of 34 CpGs for the MHP diet and 14 out of 20 CpGs for the LF diet. With these CpG sites, a multiple linear regression was performed for each of the diets, using the percentage of BMI loss as the dependent variable and methylation of the CpG sites as the independent variable. Then, those CpG sites that presented a *p* < 0.19 in the latter multiple linear regression model were included in the next analytical step, which was the sub-scores calculation (see Section 2.2). Also, collinearity was verified (>1, estat vif, Stata), eliminating the CpGs that presented multicollinearity (*n* = 3).

With these requirements, 15 CpGs for the MHP diet and 11 CpGs for the LF diet were identified. Table 5 shows the multiple linear regression values of the selected CpG sites for the MHP diet prediction model, and Table 6 shows those for the LF diet prediction model (Figure 3). The CpGs correlated with BMI loss for each diet do not show overlap.

### 3.3. Design of Weighted Sub-Scores That Contain the CpG Sites of Each Diet and the Calculation of the Total Methylation Score for the Prediction Model

Weighted sub-scores were made for each diet, using the sum of the previously selected CpG sites and multiplying them by the beta coefficients obtained in each of the multiple linear regressions of the MHP diet and the LF diet (Table 7).

To obtain a total score for each individual that would allow to be included as a term for the interaction with the diet variable, the MHP diet sub-score was subtracted from the LF diet sub-score, as shown in Table 7.

A linear mixed effect model was used to predict, based on the total methylation score of each individual, which diet will be the best for the volunteers based on the greatest percentage of BMI loss. Therefore, a linear mixed effect model was designed with the percentage of BMI loss as the dependent variable and total score, diet (MHP/LF), and the interaction term between the total score and the diet as a fixed effect. Moreover, the IDs of the participants were included as a random effect. The model was adjusted for sex and age. Table 8 shows the independent variables, the beta coefficient with the standard error, and the *p*-value. As it can be seen in the table, the model is not affected when adjusting for sex and age, since none of these variables showed statistical significance.

### 3.4. Representation of the Prediction Model

**Figure 4:**
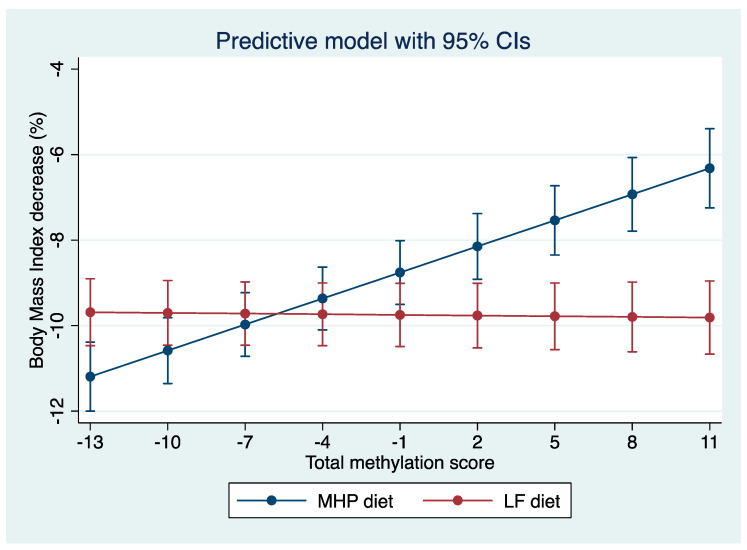
Prediction model of the percentage of BMI loss according to the total methylation score of each individual. “Y” axis: percentage of BMI loss and “X” axis: methylation score. According to the total score, it can be predicted with which diet the greatest percentage of BMI is achieved for each individual.

Based on the linear mixed effect model, a “diet x total score” interaction graph was made showing the marginal percentage of BMI loss for each diet. As shown in Figure 4, where the “X” axis represents the total methylation score and the “Y” axis shows the percentage of BMI loss, the percentage of BMI loss with each diet is estimated (MHP in blue and LF in red) according to the methylation score presented by the subjects before starting the intervention. Thus, for methylation score values between −10 and −1, the error bars that predict the percentage of BMI loss for each diet overlap, indicating that both diets are effective in losing similar BMI values. It should be noted that, between −13 and −10, the overlap is less. Conversely, when an individual has a score that is between 2 and 11, the error bars separate, suggesting that the methylation score is effective in predicting the type of diet that is the best for weight loss in that individual.

The statistical differences between the predictions of the percentage of BMI loss with both diets for each individual were analyzed using the “Z”-test, which involves the standard errors of each estimation of the score. A significant *p* (<0.05) allows prescribing the most appropriate diet for each individual. However, if the *p* is not significant (*p* > 0.05), the two diets have a similar effect, so they can be prescribed interchangeably.

**Figure 5:**
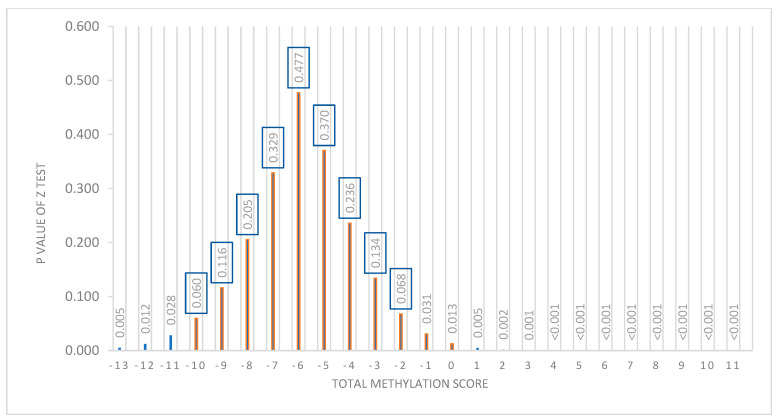
Representation of the distribution of the *p*-value of the Z-test with respect to the total methylation score. “X” axis shows the total score. “Y” axis shows the *p*-value of the Z-test. A “*p*” value <0.05 was considered significant. From −10 to −1 of the score, a nonsignificant *p* is presented.

With this test, it was observed that, of the 201 participants, we could not predict which diet was better in 126 participants, but 75 participants could be advised on one diet being better than the other based on BMI loss predictions. As shown in Figure 5, in which the “X” axis represents the total score and the “Y” axis shows the *p*-value obtained in the Z-test, the nonsignificant *p*-values (>0.05) are between the total methylation score values of −10 and −2. This is the population for which it is not possible to predict which diet is better (*n* = 126). However, for the individuals who have a score between −13 and −11 or between −1 and +11 (*n* = 75), a type of diet for losing weight can be recommended to them according to their baseline methylation score value (*p* < 0.05).

**Figure 6:**
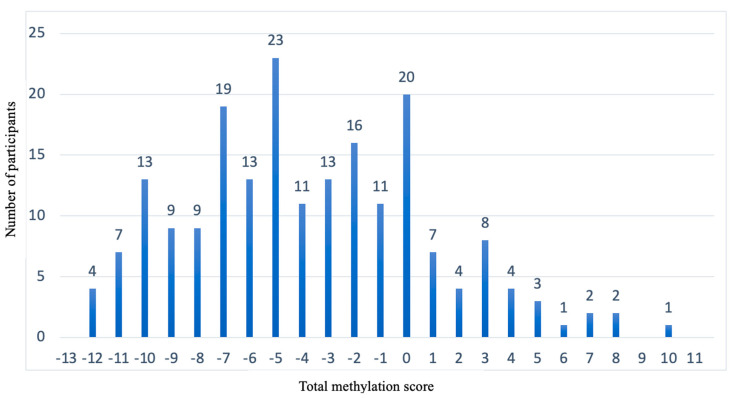
Representation of the population distribution with respect to the total methylation score. “X” axis shows the total methylation score. “Y” axis shows the number of participants.

Figure 6 shows how the 201 participants are distributed (“Y” axis) with respect to the total methylation score (the “X” axis). The population (*n* = 126) that presents total methylation score values between −10 and −2 is the population for which we cannot predict which diet is better for BMI loss.

The baseline anthropometric and biochemical data of the population whose diet could be predicted to be better with the model were compared with the data of the population whose diet could not be predicted. It was verified that the prediction was due to the percentage of methylation, since there were no differences in any of these variables between the two population groups (*p* < 0.05) (Supplementary Table S1).

### 3.5. Information on the Methylation Sites Selected for the Prediction Model

Table 9 and Table 10 show the location of the CpGs selected for the MHP diet and the LF diet, respectively. The chromosome and its coordinates, the gene, and the region within the gene in which it is found, which is information obtained by the array “Illumina”, are indicated in the tables. In the case of CpGs found in the intergenic zone, the closest gene and its distance from the CpGs were searched in the UCSC Genome Browser on Human (GRCh37/hg19). In addition, the polymorphisms associated with the selected methylation sites and their minor allele frequency for both diets are shown in the Supplementary Tables (Supplementary Table S2 for the MHP diet and Supplementary Table S3 for the LF diet).

### 3.6. Biological Role of the Genes Related to the CpG Sites Selected for the Prediction Model

**Figure 7:**
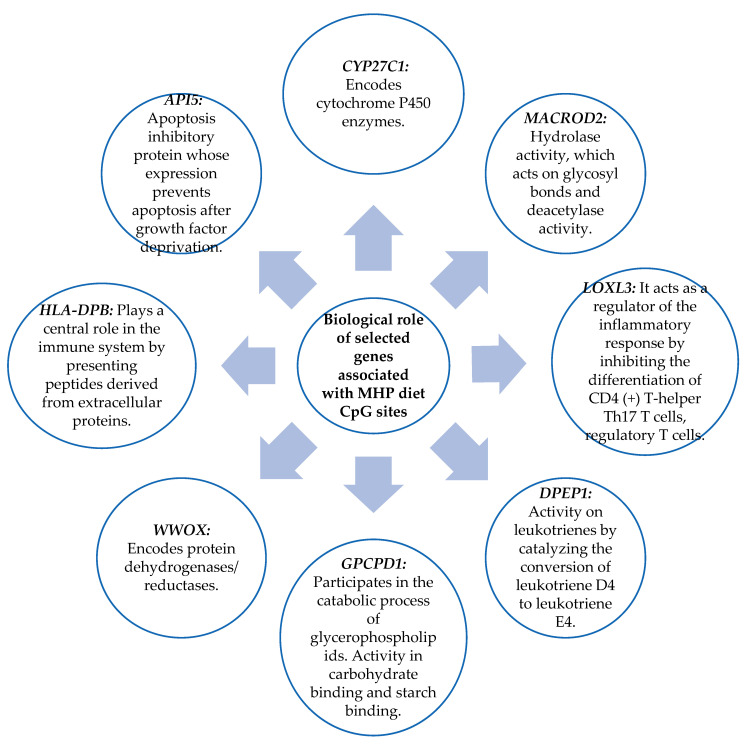
Biological role of genes that have functions in metabolism, the immune system, and cell function and that contain the CpG sites selected for the MHP diet prediction model.

**Figure 8:**
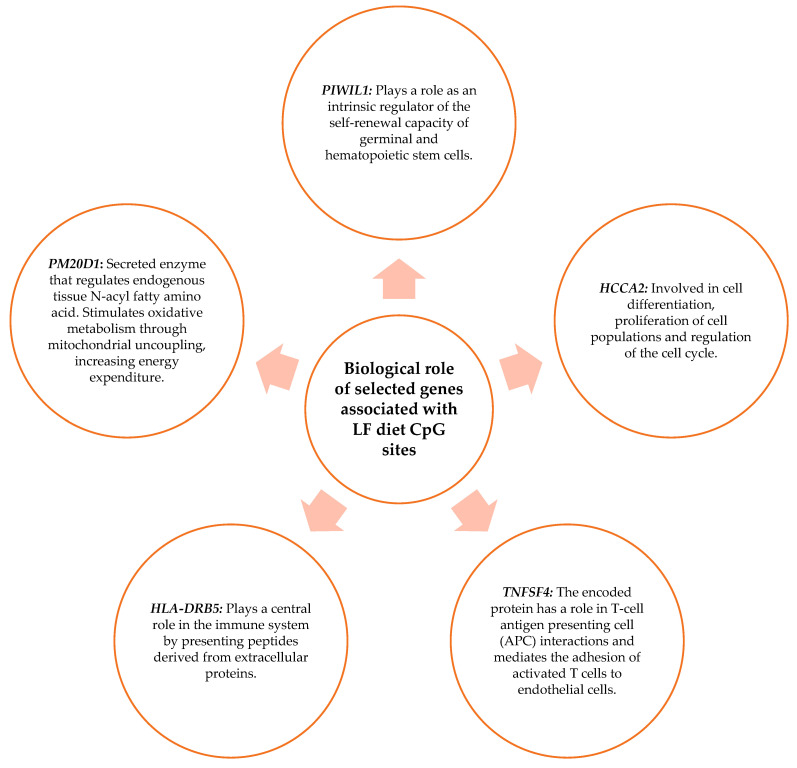
Biological role of genes that have functions in metabolism, the immune system, and cell function and that contain the CpG sites selected for the LF diet prediction model.

The biological role of the genes related to the CpG sites obtained in Section 3.2 was studied. The biological functions of the genes were searched in the “GeneCards the human gene database”. Those that present functions in metabolism, in the immune system, or cellular function were chosen. As shown in Figure 7, the genes chosen for the MHP diet were *CYP27C1*, *MACROD2*, *LOXL3*, *DPEP1*, *GPCPD1*, *WWOX*, *HLA DPB*, and *API5*. Figure 8 shows the genes selected for the LF diet: *PIWIL*, *HCCA2*, *TNFSF4*, *HLA-DRB5*, and *PM20D*.

## 4. Discussion

In this research project, the association between DNA methylation in the blood and the reduction of the BMI percentage with an intervention with two types of diets, a moderately high-protein diet (MHP) and a low-fat diet (LF), was studied in a Spanish population. Likewise, the CpG sites which basal methylation was associated with the reduction of the percentage of BMI after 4 months of dietary intervention were identified and then used to construct a total methylation score that was included in a model together with the two types of diets, and it adequately predicted the percentage of BMI loss.

### 4.1. Methylation Analyzed in Blood Samples Showing Association with the BMI

Thirty-four CpG sites for the MHP diet and twenty CpG sites for the LF diet were identified in blood and were associated with the percentage of BMI loss. This relationship of methylation sites with BMI has been observed in previous studies, such as in a clinical trial in a multiethnic Asian population that identified the methylation of 116 CpG sites associated with BMI and the methylation of 8 CpG sites that were associated with waist circumference [27]. On the other hand, in a longitudinal experimental study that was performed in a US population, they associated more than 300 CpG sites in blood with the BMIs of 480 adults [28]. Similarly, in a cross-sectional study linking blood methylation in obesity-related genes, they affirmed the association between multiple CpG sites and the BMI [29]. The CpG sites in these aforementioned studies differed from those identified with this research, so it should be noted that CpG sites related to BMI may vary according to ethnicity, population characterization, and BMI selection methods; however, it can be said that methylation contributes to determining BMI.

### 4.2. Genes Related to CpG Sites Associated with the Percentage of BMI Loss for the MHP Diet and for the LF Diet

Another finding of this investigation is the biological role of the genes with the methylation sites in the blood associated with the percentage of BMI loss for both diets. Of these genes, those with functions in the cell cycle, the immune system, and metabolism were highlighted.

In an experimental study, they analyzed the epigenetic marks related to obesity and studied the blood methylation of the *MACROD2* gene, which was positively associated with BMI levels, considering that the methylation of this gene may be involved in the development of obesity [30]. In contrast, in the present investigation, blood methylation of the *MACROD2* gene showed a positive association with the percentage of BMI loss. However, we analyzed the methylation of *MACROD2* in the body region, while the other study analyzed it in the TSS200 region. In a review study, methylation and gene expression changes were investigated in subcutaneous adipose tissue of pregnant women with gestational diabetes, in which they found that the *HLA-DRB5* gene was hypermethylated. The authors concluded that changes in the methylation of this gene may represent adaptive fetal and placental responses to glucose intolerance [31]. However, in the present investigation, we found that blood methylation of the *HLA-DRB5* gene was related to the reduction of the percentage of BMI after a nutritional intervention. Likewise, in a clinical trial, other authors analyzed the effect of methylation in the *WWOX* gene on osteosarcoma cell proliferation. This study was performed in bone tissue affected by osteosarcoma and compared with healthy bone tissue, and it demonstrated that *WWOX* methylation levels were increased in patients with osteosarcoma [32].

Another study observed that in vitro gastric cancer cell lines infected with *H. pylori* showed increased methylation in the *WWOX* gene [33]. In our study, the blood methylation of the *WWOX* gene showed a negative correlation with the decrease in BMI percentage. The subjects who had higher methylation levels showed lesser BMI percentage decreases. Therefore, it seems that hypermethylation of this gene is associated with negative changes in metabolic health.

It is important to highlight that, in the present investigation, the study of methylation was analyzed in blood, and in some previously mentioned studies, the methylation of these same genes was studied in target tissues, which demonstrates that blood tissue can serve as a rather similar reflection of methylation status, as it has been analyzed in some studies that compared methylation in a target tissue versus identifying methylation in blood, in which methylation data were considered to be more specific if performed in the tissue of interest for the investigation. But blood is a valid option that can provide fairly accurate information on the methylation status whenever adjustments are made to the blood cell composition, since it has the advantage of being a more accessible tissue, which can be taken routinely [34,35].

### 4.3. Prediction Model

Currently, the usual management for overweight and obesity is done by calculating energy expenditure, establishing daily nutritional requirements, and performing caloric restriction, in addition to giving recommendations for lifestyle changes specific to each individual [36,37]. The main limitation of this routine management is that it does not take into account the variability of each individual’s response to these interventions. Therefore, we designed a prediction model based on methylation data from the studied population that predicts the percentage of BMI loss with the MHP diet and the LF diet. With this pioneer approach, we were able to predict, for many individuals, the most appropriate diet for losing a higher percentage of BMI in a dietary intervention for obesity or overweight. This could also help to increase the adherence to the intervention. Linear mixed models were used to model the BMI loss, which included the interaction term between the methylation score biomarker and the categorical variable encoding the two diets (MHP and LF). This linear mixed effect model was designed with the percentage of BMI loss as the dependent variable and the total score, diet (MHP/LF), and the interaction term between the total score and the diet as a fixed effect. Since the biomarker–diet interaction term is essential to deduce the relevant effects of the individualized treatment, we used the total methylation score biomarker resulting from subtracting the methylation sub-scores obtained for each diet (see Table 7). Finally, the model was adjusted for sex and age.

### 4.4. BMI Percentage Loss Prediction Model Based on the MHP and LF Diets’ Methylation Data

The model that predicts the percentage of BMI loss was made with a total methylation score that was constructed considering the MHP diet methylation score, in which the 15 CpG sites that were better associated with the reduction of BMI percentage were selected and 11 CpG sites for the LF diet. With this model, it was possible to predict with which diet the highest percentage of BMI would be lost for 75 participants, corresponding to 37.3% of the total population of this study. We consider that this total methylation score could be used to predict which diet is more appropriate for each individual. If not significant, the predictive model indicates that these subjects would lose a similar percentage of BMI with both diets. In this case, and in order to increase the adherence to the treatment, the individuals could choose by dietary preference.

The potential of DNA methylation to predict BMI loss has been described before, as demonstrated in a study in which a prediction score was performed based on 83 CpG sites that was associated with BMI; the result they obtained was a prediction that represented 29% of the variation in BMI in the population they studied [38]. This prediction model of the percentage of BMI loss had no similarity in CpG sites and genes related to the CpG sites that we identified for both diets. This may be due to the differences in the investigations, such as different methods and selection parameters of the CpG sites. Another important point is the variability in the data due to different sizes, population characteristics, and experimental protocols. These are some of the reasons that may lead to differences in the results between studies.

To conclude, it is important to mention that this pioneer prediction model can be improved by adding other information, because the use of other variables for the prediction of the decrease in anthropometric measurements with a dietary intervention has been evidenced. For example, a useful weight loss prediction model using gut microbiota data and urine metabolites in a nutritional intervention has been recently published [39]. The use of polymorphisms as useful biomarkers to predict weight loss with a diet has also been described in previous research [40,41]. In fact, the approach used in the present study is similar to that employed in a previous publication of our group that used SNPs to predict the responses to the same weight loss diets [42]. These studies evidence that the use of other variables, such as genetics, microbiota, and metabolomics, could be useful in predicting the reduction of anthropometric measures or the response to an intervention. Therefore, in future studies, it would be interesting to integrate these variables into the epigenetic model that has been carried out in this research. It is possible that a prediction model that includes epigenetic, genetic, and/or metagenomic data will improve and may provide advantages for their implication in precision nutrition.

### 4.5. Strengths and Limitations

One of the main strengths of this pilot research is that it was carried out within the framework of a randomized clinical study in which more than 200 people were characterized. A methylation array was used to analyze around 850,000 methylation sites in each individual at baseline. Multiple methylation sites were identified as associated with the reduction in BMI percentage after the intervention with the two types of diets, thus demonstrating the impact that epigenetics has on the response and regulation of anthropometric measures and suggesting that epigenetic markers can be very useful in the precision dietary treatment of obesity. Furthermore, although a pilot study, it was a robust study, since the association of CpG sites with the dietary intervention response was not affected by potential confounding factors, as the model was adjusted for age, sex, and cell composition.

The designed epigenetic model successfully predicted the percentage of BMI loss with each of the two diets (MHP and LF). For this model, blood was used as the study tissue, which is an accessible, easy, minimally invasive tissue. Therefore, the designed methylation score can be used as biomarkers in the future using blood samples. On the other hand, with the results obtained, future research can be developed, and the model can also allow the integration of new variables for improvement.

It is important to mention that the information obtained from the methylation data of these subjects refers to the methylation situation at that moment and of the tissue that was studied, but this information may vary with time and the tissue analyzed.

Another limitation of this pioneer study was the expression of the genes in which the methylation sites identified in this research were located, as it was not analyzed.

It would be interesting to know if the changes in methylation have any real influence on the expression levels. It should be taken into account that this research was carried out in a Spanish population, mostly of Caucasian origin, so the data obtained cannot be extrapolated to a demographically different population. It is important to point out that several studies suggest that epigenetic marks are dependent on race, origin, and many lifestyle factors, including perinatal factors, and there are many factors that influence the degree of individual methylation, so that small differences in methylation can be found between individuals. As DNA methylation somewhat shows a maternal inheritance, another limitation is that we did not have twins in the study.

On the other hand, there are technological limitations that can influence methylation values, as they are dependent on the equipment used; reagents; and sample handling (blood preservation, cell isolation, DNA extraction, bisulfite treatment, etc.). For this reason, it is difficult to compare between different studies.

## 5. Conclusions

This pioneer research demonstrates that DNA methylation is an individual characteristic that can be used to have greater precision in the nutritional treatment of BMI reduction. The model designed based on the methylation information through the linear mixed effect model allows predicting the percentage of BMI loss and could be useful in determining which diet is more adequate for weight loss for each individual.

## Figures and Tables

**Table 1:** Baseline anthropometric and biochemical data from the groups with a moderately high-protein (MHP) and a low-fat (LF) diet.

	MHP (*n* = 93)	LF (*n* = 108)	*p*
Gender (male/female) *n* (% male)	28 (30%)	33 (31%)	0.945
Age	52 ± 1	54 ± 1	0.277
BMI (kg/m^2^)	31.2 ± 0.3	32.1 ± 0.3	0.062
Body weight (kg)	86.6 ± 1.4	88.7 ± 1.1	0.256
Waist circumference (cm)	101 ± 1	102 ± 0	0.463
Hip circumference (cm)	111 ± 1	112 ± 1	0.225
Lean mass dxa (g)	47585 ± 1009	48646 ± 939	0.442
Fat mass dxa (g)	36190 ± 734	37250 ± 752	0.317
Visceral fat mass dxa (g)	1362 ± 91	1486 ± 79	0.301
Diastolic pressure (mmHg)	78. ± 1	79 ± 1	0.399
Systolic pressure (mmHg)	130 ± 2	128 ± 1	0.511
Total energy (Kcal)	1509 ± 24.1	1514 ± 19.5	0.863
Glucose (mmol/L)	5 ± 0.5	5 ± 0.1	0.317
Insulin (mU/l)	7.7 ± 0.5	8.1 ± 0.4	0.703
Leptin (ng/mL)	34.2 ± 2.4	38.1 ± 2.9	0.325
Adiponectin (μg/mL)	11.1 ± 0.4	11.5 ± 0.4	0.577
HOMA-IR	1.8 ± 0.1	1.9 ± 0.1	0.726
Cholesterol (mg/dL)	214 ± 3	218 ± 3	0.534
HDL-cholesterol (mg/dL)	53 ± 1	55 ± 1	0.301
Triglycerides (mg/dL)	98 ± 4	103 ± 5	0.505
LDL-c (mg/dL)	1400 ± 3	141 ± 3	0.911
ox-LDL (mg/dL)	44 ± 1	46 ± 1	0.303
Alt (IU/L)	24.3 ± 1.7	23.1 ± 1.1	0.506
Ast (IU/L)	22.5 ± 1.2	21.5 ± 0.6	0.468
Uric acid (mg/dL)	5.1 ± 0.1	5.2 ± 0.1	0.433
C- Reactive protein (mg/L)	2.7 ± 0.2	3.1 ± 0.3	0.551
TNF-α (pg/mL)	0.9 ± 0.3	0.8 ± 0.3	**0.006**

Values correspond to the mean ± SEM. BMI: body mass index, HOMA-IR: insulin resistance index. HDL: high-density lipoprotein, LDL: low-density lipoprotein, ox-LDL: oxidized low-density lipoprotein, Alt: alanine aminotransferase, Ast: aspartate aminotransferase, and TNF-α: tumor necrosis factor alpha. *p* < 0.05 highlighted in bold was considered statistically significant. The mean of the sex was calculated with chi-square. The *p*-value of the comparison of the baseline means between the MHP diet and LF was calculated using the Student’s *t*-test for independent samples.

**Table 2:** Anthropometric and biochemical changes that occurred with the interventions with each of the two diets (MHP and LF) after 4 months and the differences between them.

MPH Diet (*n* = 93)			LF Diet (*n* = 108)		Comparison of the Differences between the MHP Diet and the LF Diet	
	Mean ± SEM	*p* ^1^	Mean ±SEM	*p* ^2^	Mean ± SEM	*p* ^3^
**Δ BMI (kg/m^2^)**	−3.1 ± 0.1	**<0.001**	−3.2 ± 0.1	**<0.001**	0.2 ± 0.1	0.261
**Δ Body weight (kg)**	−8.4 ± 0.3	**<0.001**	−9.1 ± 0.3	**<0.001**	0.5 ± 0.5	0.272
**Δ Waist circumference (cm)**	−8.9 ± 0.4	**<0.001**	−9.4 ± 0.3	**<0.001**	0.5 ± 0.6	0.351
**Δ Hip circumference (cm)**	−6.2 ± 0.3	**<0.001**	−6.7 ± 0.3	**<0.001**	0.5 ± 0.5	0.295
**Δ Lean mass dxa (g)**	−1221 ± 130	**<0.001**	−1662 ± 140	**<0.001**	441 ± 193	**0.023**
**Δ Fat mass dxa (g)**	−6841 ± 357	**<0.001**	−7103 ± 299	**<0.001**	262 ± 463	0.572
**Δ Visceral fat mass dxa (g)**	−454 ± 42	**<0.001**	−487 ± 36	**<0.001**	33 ± 55	0.555
**Δ Diastolic pressure (mmHg)**	−3.7 ± 0.9	**<0.001**	−4.1 ± 1.1	**<0.001**	0.2 ± 1.4	0.863
**Δ Systolic pressure (mmHg)**	−12.3 ± 1.3	**<0.001**	−10.1 ± 1.1	**<0.001**	−2.1 ± 1.7	0.213
**Δ Total energy (Kcal)**	415 ± 16	**<0.001**	428 ± 11	**<0.001**	−13 ± 21	0.521
**Δ Glucose** **(** **mmol/L)**	−0.7 ± 0.5	**<0.001**	−0.2 ± 0.4	**<0.001**	−0.5 ± 0.5	0.296
**Δ Insulin (mU/l)**	−2.8 ± 0.4	**<0.001**	−2.6 ± 0.3	**<0.001**	−0.2 ± 0.6	0.694
**Δ Leptin (ng/mL)**	−16.2 ± 2.1	**<0.001**	−19.1 ± 1.9	**<0.001**	2.9 ± 2.8	0.301
**Δ Adiponectin (μg/mL)**	0.4 ± 0.2	**0.047**	0.1 ± 0.2	0.748	0.3 ± 0.3	0.311
**Δ HOMA-IR**	−0.7 ± 0.1	**<0.001**	−0.7 ± 0.1	**<0.001**	−0.6 ± 0.1	0.701
**Δ Cholesterol (mg/dL)**	−17.8 ± 2.5	**<0.001**	−22.1 ± 2.5	**<0.001**	4.2 ± 3.6	0.247
**Δ HDL-c (mg/dL)**	−2.7 ± 0.7	**0.001**	−4.8 ± 0.8	**<0.001**	2.1 ± 1.1	**0.059**
**Δ Triglycerides (mg/dL)**	−19.7 ± 4.1	**<0.001**	−15.1 ± 3.6	**<0.001**	−4.6 ± 5.4	0.389
**Δ LDL-c (mg/dL)**	−11.1 ± 2.1	**<0.001**	−14.2 ± 1.9	**<0.001**	3.1 ± 2.8	0.281
**Δ ox-LDL (mg/dL)**	−8.1 ± 0.9	**<0.001**	−8.6 ± 1.2	**<0.001**	0.5 ± 1.6	0.722
**Δ Alt (IU/L)**	−3.9 ± 1.8	**0.003**	−3.6 ± 0.9	**<0.001**	−0.3 ± 1.9	0.867
**Δ Ast (IU/L)**	−0.6 ± 1.6	0.686	−1.0 ± 0.6	0.074	0.4 ± 1.6	0.787
**Δ Uric acid (mg/dL)**	−0.1 ± 0.1	**0.013**	−0.2 ± 0.1	**<0.001**	0.1 ± 0.1	0.087
**Δ C- Reactive protein (mg/L)**	−0.9 ± 0.2	**<0.001**	−1.0 ± 0.2	**<0.001**	0.1 ± 0.3	0.738
**Δ TNF** **-α** **(pg/mL** **)**	0.03 ± 0.01	0.079	0.013 ± 0.02	0.511	−0.04 ± 0.02	0.098

Values correspond to the baseline mean and mean changes after caloric restriction treatment ± SEM. BMI: body mass index. HOMA-IR: insulin resistance index. HDL: high-density lipoprotein, LDL: low-density lipoprotein, ox-LDL: oxidized low-density lipoprotein, and Alt: alanine aminotransferase. Ast: aspartate aminotransferase. TNF-α: tumor necrosis factor alpha. *p* < 0.05 highlighted in bold was considered statistically significant. ^1^ Values from the comparison of the baseline and post-intervention means of the group assigned with the MHP diet; ^1^ *p*-value of the changes was calculated using the Student’s *t*-test for dependent samples. ^2^ Values from the comparison of the baseline and post-intervention means of the group assigned to the LF diet; ^2^ *p*-value of the changes was calculated using Student’s *t*-test for dependent samples. ^3^ Mean values of post-intervention changes in the MHP and LF diets; ^3^ *p*-value of the comparison of post-intervention changes of the MHP and LF diets was calculated using the Student’s *t*-test for independent samples.

**Table 3:** Spearman’s correlations between methylation of the significant CpG sites (*p* < 0.05) and percent of BMI loss with the MHP diet.

CpG Sites	Annotated Gene	Rho	*p*
cg00124993	*MIR886*	−0.212	0.041
cg00308130		−0.218	0.035
cg01097406		−0.238	0.022
cg03447554		−0.243	0.019
cg04481923	*MIR886*	−0.229	0.027
cg06478886		−0.212	0.041
cg06536614	*MIR886*	−0.211	0.042
cg07104639	*MACROD2*	0.207	0.046
cg07782112		−0.208	0.045
cg08745965	*MIR886*	−0.222	0.032
cg09768983		0.224	0.031
cg10841563		−0.233	0.024
cg11460778		0.217	0.036
cg14317533		−0.214	0.039
cg15263617	*GPCPD1*	0.239	0.021
cg15837280		−0.265	0.010
cg17052675		−0.245	0.018
cg17764313	*MCM2;MCM2*	−0.237	0.022
cg18678645	*MIR886*	−0.207	0.046
cg18797653	*MIR886*	−0.210	0.043
cg19053046	*HLA-DPB1*	0.221	0.033
cg19148731	*LOXL3*	−0.214	0.039
cg19504605	*ZFP41*	−0.204	0.049
cg20315590	*HMCN1*	−0.215	0.038
cg20684491		0.301	0.003
cg21054447		0.207	0.046
cg22355889	*ELMOD1;LOC643923;ELMOD1*	0.207	0.046
cg23377942	*WWOX;WWOX*	−0.206	0.048
cg23899408	*HOOK2;HOOK2*	−0.224	0.031
cg24433124		−0.223	0.031
cg24658778	*SYNE1;SYNE1*	−0.269	0.009
cg25340688	*MIR886*	−0.227	0.028
cg26896946	*MIR886*	−0.239	0.021
cg27149073	*SDHAP3*	0.222	0.032

Spearman’s correlations between methylation of the significant CpG sites and the percentage of BMI loss of the 201 subjects. *p*-value < 0.05 was considered significant, resulting in *n* = 34 CpG sites for the MHP diet.

**Table 4:** Spearman’s correlations between methylation of the significant CpG sites (*p* < 0.05) and percent of BMI loss with the LF diet.

Sitios CpG	Annotated Genes	Rho	*p*
cg00481382	*NEDD1;NEDD1;NEDD1;NEDD1*	0.250	0.009
cg03188948		−0.192	0.046
cg04346459	*NFYA;NFYA;LOC221442*	0.204	0.034
cg07167872	*PM20D1*	0.196	0.042
cg11193064	*SMAD6;SMAD6*	−0.196	0.042
cg14050976		0.203	0.035
cg14222729		−0.236	0.014
cg14893161	*PM20D1;PM20D1*	0.194	0.043
cg15011943	*HLA-DRB5*	0.205	0.033
cg15572235		0.234	0.014
cg15695738		0.190	0.049
cg15837280		0.190	0.048
cg16078649	*RNF39;RNF39*	−0.262	0.006
cg16600909		0.220	0.022
cg17035276		−0.216	0.024
cg18493115	*HCCA2;KRTAP5-4*	−0.231	0.016
cg19424457	*PIWIL1;PIWIL1*	−0.210	0.029
cg20057198		−0.251	0.009
cg24433124		0.220	0.022
cg26967960	*CAV3;CAV3*	0.287	0.003

Spearman’s correlation of CpG sites with the percentage of BMI loss. *p*-value < 0.05 was considered significant, resulting in *n* = 20 CpG sites for the LF diet.

**Table 5:** Multiple linear regression for MHP diet prediction model showing an association between methylation of the CpG sites and BMI difference (*p* < 0.19).

MHP Diet (*n* = 93 Participants)			
CpG Sites	Beta Coefficient	SEM	*p*
cg22355889	9.818	2.3	<0.001
cg24433124	−5.813	1.7	0.002
cg14317533	−7.578	2.3	0.002
cg07104639	6.259	2.14	0.005
cg19148731	−7.880	2.2	0.001
cg01097406	−5.982	1.6	0.001
cg15263617	8.975	2.8	0.002
cg23377942	−4.191	2.3	0.075
cg25340688	−5.795	1.8	0.003
cg19053046	5.080	2.6	0.055
cg11460778	4.222	1.7	0.016
cg03447554	−4.080	1.9	0.035
cg09768983	3.989	2.5	0.125
cg20684491	4.803	1.9	0.015
cg20315590	−6.713	2.8	0.022

Multiple linear regression model using the percentage of BMI loss as the dependent variable and the CpG sites for the MHP diet as independent variables. SEM: standard error. Beta values represent changes in results for the increasing number of BMI percent loss units.

**Table 6:** Multiple linear regression for the LF diet prediction model showing an association between methylation of the CpG sites and BMI difference (*p* < 0.19).

LF Diet (*n* = 108 Participants)			
CpG Sites	Beta Coefficient	SEM	*p*
cg15572235	7.570	2.8	0.010
cg19424457	−8.048	3.1	0.011
cg18493115	−6.267	3.1	0.048
cg00481382	5.789	2.8	0.047
cg16600909	5.380	3.5	0.129
cg15837280	5.830	3.0	0.058
cg03188948	−4.308	2.4	0.081
cg16078649	−4.163	2.6	0.121
cg15011943	4.649	3.3	0.169
cg07167872	3.986	1.7	0.027
cg14222729	−3.407	2.5	0.187

Linear regression model using the percentage of BMI loss as the dependent variable and the CpG sites for the LF diet as independent variables. SEM: standard error. Beta values represent changes in results for the increasing number of BMI percent loss units.

**Table 7:** Design of weighted sub-scores for the diet MHP, LF, and total score.

Score	Calculating Formula
Sub-score MHP Diet	(cg22355889 * 9.82) + (cg24433124 * −5.81) + (cg14317533 * − 7.58) + (cg07104639 * 6.25) + (cg19148731 * −7.88) + (cg01097406 * −5.98) + (cg15263617 * 8.97) + (cg23377942 * −4.19) + (cg25340688 * −5.79) + (cg19053046 * 5.08) + (cg11460778 * 4.22) + (cg03447554 * −4.08) + (cg09768983 * 3.98) + (cg20684491 * 4.80) + (cg20315590 * −6.71)
Sub-score LF Diet	(cg15572235 * 7.57) + (cg19424457 * −8.04) + (cg18493115 * −6.26) + (cg00481382 * 5.78) + (cg16600909 * 5.38) + (cg15837280 * 5.83) + (cg03188948 * −4.30) + (cg16078649 * −4.16) + (cg15011943 * 4.64) + (cg07167872 * 3.98) + (cg14222729 * −3.40)
Total Methylation Score	Sub-score MHP − Sub-Score LF

**Table 8:** Linear mixed effect model for the prediction of the percentage of BMI loss.

Prob > Chi-Square ≤ 0.001 Participants (*n* = 201)	Percentage of BMI Loss	
Independent Variable	Beta Coefficient ± SEM	*p*-Values Z-Test
Age	−0.001 ± < 0.001	0.943
Sex	−0.006 ± 0.1	0.954
Diet (MHP or LF)	−1.201 ± 0.1	<0.001
Total methylation score	0.202 ± 0.01	<0.001
Diet## total methylation score	−0.208 ± 0.02	<0.001
Cons	−7.952 ± 0.6	<0.001

Dependent variable percentage of BMI loss, fixed effect variable diet, total score, and interaction of the diet and total score. Adjusted for age and sex. Diet## total score indicates the interaction between both variables. SEM: standard error.

**Table 9:** Information from the 15 methylation sites associated with the MHP diet selected for the prediction model.

CpG Sites	Chromosome	Map INFO	Gene ^1^	Gene Region ^2^
cg22355889	11	107461585	*ELMOD1*	TSS1500
cg07104639	20	15125595	*MACROD2*	Body
cg19148731	2	74780229	*LOXL3*	5′UTR
cg15263617	20	5574362	*GPCPD1*	Body
cg23377942	16	79042805	*WWOX*	Body
cg25340688	5	135416398	*MIR886*	TSS200
cg19053046	6	33048254	*HLA-DPB1*	Body
cg20315590	1	186003041	*HMCN1*	Body
cg20684491	1	25596433	*RSRP1*	IGR (2476)
cg11460778	1	145385299	*NBPF10*	IGR (24,744)
cg03447554	11	43094025	*API5*	IGR (239,480)
cg09768983	4	183935060	*DCTD*	IGR (143,816)
cg01097406	16	89675127	*DPEP1*	IGR (−4589)
cg24433124	6	30755968	LINC00243	IGR (−24,675)
cg14317533	2	127886316	*CYP27C1*	IGR (−55,096)

Information from methylation sites of the MHP diet prediction model by using the “Illumina MethylationEPIC array”. The location on the chromosome, the related gene, and the region in which each CpG is found were analyzed for each methylation site. Gene ^1^: information by Illumina, except for CpG sites located in intergenic regions (IGR), where a search was performed in “UCSC Genome Browser on Human (GRCh37/hg19)” for the closest gene. Gene region ^2^: 5′UTR: 5′ untranslated, TSS1500: transcription start site 1500, TSS200: transcription start site 200, body: body, and IGR: intergenic region. In the case of the IGR, the distance between the CpGs and the closest gene is shown in parentheses.

**Table 10:** Information from the 11 methylation sites associated with the LF diet selected for the prediction model.

CpG Sites	Chromosome	Map INFO	Gene ^1^	Gene Region ^2^
cg19424457	12	130822308	*PIWIL1*	TSS200
cg18493115	11	1643842	*HCCA2*	Body
cg00481382	12	97304412	*NEDD1*	5′UTR
cg16078649	6	30039466	*RNF39*	Body
cg15011943	6	32493917	*HLA-DRB5*	Body
cg07167872	1	205819463	*PM20D1*	TSS200
cg15572235	7	5183992	*RBAK*	IGR (98,540)
cg16600909	1	173145001	*TNFSF4*	IGR (−7869)
cg15837280	5	135415258	*TGFBI*	IGR (50,674)
cg03188948	7	1209495	*ZFAND2A-DT*	IGR (9367)
cg14222729	2	731215	*TMEM18*	IGR (63,242)

Information from the methylation sites of the LF diet prediction model by using the “Illumina MethylationEPIC array”. The location on the chromosome, the related gene, and the region in which each CpG is found was analyzed for each methylation site. Gene ^1^: information by Illumina, except for CpG sites located in intergenic regions (IGR), where a search was performed in “UCSC Genome Browser on Human (GRCh37/hg19)” for the closest gene. Gene region ^2^: 5′UTR: 5′ untranslated, TSS200: transcription start site 200, body: body, and IGR: intergenic region. In the case of the IGR, the distance between the CpG and the closest gene is shown in parentheses.

## Data Availability

Data will be available upon request by contacting the corresponding author.

## Supplementary Materials

The following supporting information can be downloaded at https://www.mdpi.com/article/10.3390/nu15245023/s1: Table S1. Baseline anthropometric and biochemical data of participants who can be given a prediction and participants who cannot be given a prediction of BMI percentage loss with the model. Table S2. Association between methylation sites and SNPs (according to “Illumina”) of the moderately high-protein diet (MHP). Table S3. Association between methylation sites and SNPs (according to “Illumina”) of the low-fat (LF) diet. Supplementary Figure S1. Scatter plots between methylation and change in BMI for each of these MHP diet CpG sites. Figure S2. Scatter plots between methylation and change in BMI for each of these LF diet CpG sites.
