# Supplementary material for: A Predictive Tool Based on DNA Methylation Data for Personalized Weight Loss through Different Dietary Strategies: A Pilot Study
Source: Nutrients. 2023 Dec 6;15(24):5023. doi: 10.3390/nu15245023 (PMC10746100; doi:10.3390/nu15245023)
Supplement: Supplementary file 1 [file nutrients-15-05023-s001.zip › nutrients-2710096-supplementary.pdf]

nutrients MDPI

Supplementary information

Supplementary Table S1. Baseline anthropometric and biochemical data of participants who can be given a prediction and participants who cannot be given a prediction of BMI percentage loss with the model.

	No Prediction (n=126)	Prediction (n=75)	Differences between both groups	P
**Gender (Men/Women)**	44 /82	17/58	27	0.068
**Age**	52.1±0.9	54.6±1.8	-2.4±1.4	0.091
**BMI (kg/ m^2)**	31.6± 0.3	31.7± 0.4	-0.03±0.5	0.940
**Weight (kg)**	88.4±1.2	86.5±1.5	-1.9±1.9	0.321
**Waist circumference(cm)**	102± 0.9	101± 1.1	-0.9±1.5	0.550
**Hip circumference (cm)**	111± 0.7	111± 0.8	-0.2±1.1	0.851
**Lean mass dxa (g)**	48844± 890.8	4700±1066.6	1843±1416.2	0.1945
**Fat mass dxa (g)**	36848±670.2	36606±861.6	242±1092.7	0.824
**Visceral fat mass dxa (g)**	1487± 79.7	1332±86.7	154±122.9	0.210
**Diastolic pressure (mmHg)**	79±1.1	79±1.2	0.5±1.6	0.727
**Systolic pressure (mmHg)**	129± 1.7	129±1.9	0.6±2.7	0.816
**Diet (MHP/LF) n(%MHP)**	61(31%)	32(18%)	29(11%)	0.429
**Total energy (Kcal)**	1530± 19.7	1482±23.8	47.7±31.4	0.130
**Glucose (mmol/L)**	5.1± 0.1	5.1±0.1	-0.1±0.1	0.874
**Insulin (mU/l)**	7.6±0.4	8.2±0.5	-0.6±0.6	0.365
**Leptin (ng/mL)**	35.5± 2.4	37.7± 3.2	-2.2±3.9	0.585
**Adiponectin (μg/mL)**	10.8±0.4	12.1±0.5	-1.2±0.7	0.087
**HOMA ir**	1.8± 0.1	2.0± 0.1	-0.2±0.1	0.392
**Cholesterol (mg/dL)**	216± 3.4	215± 4.5	0.98±5.6	0.862
**HDL-c (mg/dL)**	54± 1.2	56±1.3	-1.9±1.8	0.289
**Triglycerides(mg/dL)**	105±4.8	93± 5.2	12.2±7.4	0.105
**LDL-c(mg/dL)**	141± 3.1	140± 3.8	0.5±5.03	0.916
**LDL ox (mg/dL)**	46± 1.1	45± 1.4	0.6±1.84	0.730
**Alt (IU/L)**	22.7± 0.9	25.1±2.2	-2.3±2.1	0.266
**Ast (IU/L)**	21.7± 0.5	22.4± 1.5	-0.6±1.4	0.641
**Uric acid (mg/dL)**	5.2±0.1	5.1± 0.1	0.2±02	0.276
**C- Reactive protein (mg/L)**	3.1±0.2	2.7±0.3	0.2±04	0.524
**TNF-α (pg/mL)**	0.9± 0.3	0.8± 0.3	0.04±0.5	0.328

Data are represented as mean ± SEM.BMI: body mass index, HOMA-IR: insulin resistance index. HDL: high-density lipoprotein, LDL: low-density lipoprotein, LDL ox: oxidized low-density lipoprotein, Alt: alanine aminotransferase. Ast: aspartate aminotransferases. TNF-**α**: tumor necrosis factor alpha. P<0.05 was considered statistically significant. Sex was calculated with Chi2. #**1**The P value was calculated using Student's t test for dependent samples.

Supplementary Table S2. Association between methylation sites and SNPs according to "Illumina" of moderately high protein diet (MHP).

CpG	SNP ID	Distance SNP	MAF
cg16595667	rs184169976	33	0.000599
cg11324953	rs28656215	46	0.154353
cg26676129	rs35852004; rs540506053; rs560104704; rs529057818; rs548808509; rs143680522;rs531427124	0;3;7;16;20;23;47	0.004393;0.000200;0.000200;0.000200;0.000200;0.000799;0.000200
cg24454263	rs183951758	4	0.000998
cg00442529	rs116217107; rs563385940	14;23	0.008986;0.000200
cg10671180	rs549754257; rs552635880; rs55820639; rs534962514	36;21;14;2	0.000399;0.000200;0.084265;0.000200
cg05468370	rs571164011; rs186098253; rs34729; rs34730; rs537827400	3;14;22;30;37	0.000200;0.000200;0.464457;0.406150;0.000200
cg07710974	rs186932892	49	0.001797
cg04906352	rs183580184	11	0.0002
cg10339573	rs138636499	27	0.004193
cg19810433	rs142392033; rs145937875	0;9	0.000998;0.000399
cg10634568	rs578023542	18	0.001198
cg19723734	rs573600446; rs147796808; rs3757436; rs576770156	1;12;47;48	0.000200;0.000399;0.031150;0.000399
cg16313837	rs535070050; rs553199591; rs574915979; rs541854522	49;48;6;2	0.000599;0.000200;0.000200;0.000200
cg07322512	rs71422116; rs575096582; rs540426772	30;26;6	0.500000;0.000599;0.000200
cg02667102	rs111394148; rs72737179; rs532712448; rs547689734; rs566324190; rs536462558	39;38;25;3;2;1	0.003395;0.009385;0.000599;0.000200;0.000599;0.000200
cg01990482	rs552485357; rs569116012; rs537850661	48;39;19	0.000200;0.000200;0.000399
cg04448061	rs566782630; rs534224807	51;36	0.000200;0.000200
cg02470045	rs540571038	9	0.000399
cg20586947	rs188702396; rs576445198	47;48	0.002196;0.000998
cg03221998	rs149243986; rs573829261; rs542537744; rs144421266; rs12409509; rs34287859; rs544577633	45;39;32;31;16;7;1	0.000200;0.000200;0.000200;0.000399;0.007588;0.285942;0.000200
cg20132612	rs565444551; rs577439754	35;21	0.000200;0.000399
cg05394741	rs575125850; rs560803611	14;1	0.000200;0.000599
cg13318279	rs117684324	10	0.047724
cg02540803	rs570197812; rs535535479; rs549058182	34;7;6	0.000200;0.000399;0.000399

MAF: frequency of the minor allele in prediction.
Polymorphisms associated with methylation sites of responder and non-responder groups, recognized by the "Illumina Methylation Array".

Supplementary Table S3. Association between methylation sites and SNPs according to low-fat (LF) diet "Illumina".

CpG	SNP ID	Distance SNP	MAF
cg14625906	rs541571230; rs555295395; rs192222493; rs541283331	47;33;30;1	0.000200;0.000200;0.000200;0.000200
cg14118991	rs556501993	13	0.000399
cg10943232	rs17103037; rs531137828	15;8	0.016573;0.000200
cg19376658	rs114803340; rs183557392	35;1	0.008986;0.001398
cg05341260	rs533199065	28	0.000399
cg02042086	rs74903411; rs374838837; rs1554032; rs556210571	35;34;34;23	0.394569;0.394569;0.197284;0.000200
cg21474679	rs115798612	25	0.026957
cg12152566	rs115817593; rs188713107	28;35	0.004393;0.000599
cg03174507	rs563640063	31	0.0002
cg09994109	rs559168308; rs114748199	31;22	0.000200;0.002796
cg11082237	rs556828431	2	0.0002

MAF: minor allele frequency in prediction.
Polymorphisms associated with methylation sites of responder and non-responder groups, recognized by "Illumina Methylation Array".

Supplementary Figures S1. Scatter plots between methylation and change in BMI for each of these MHP diet CpG sites.

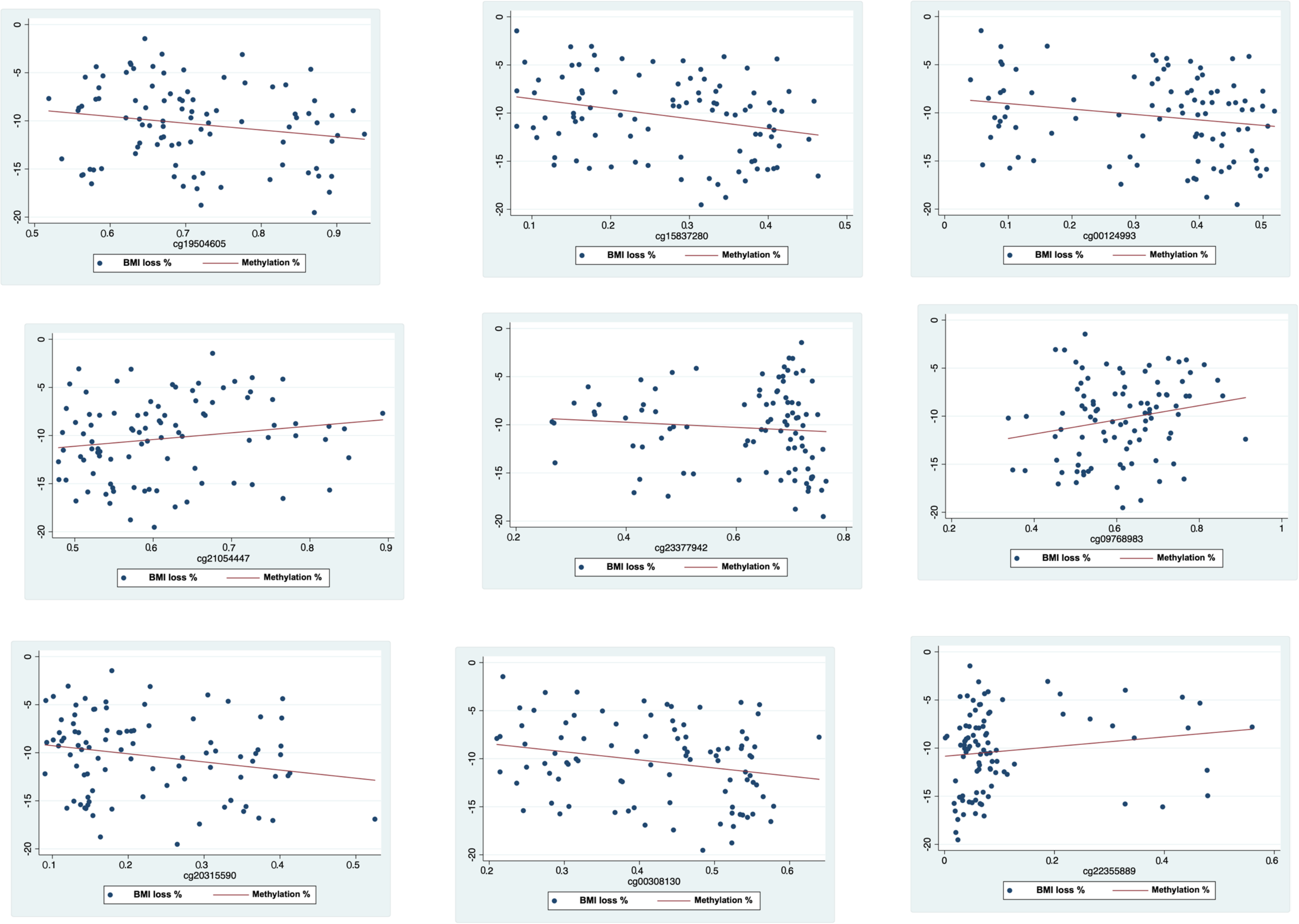

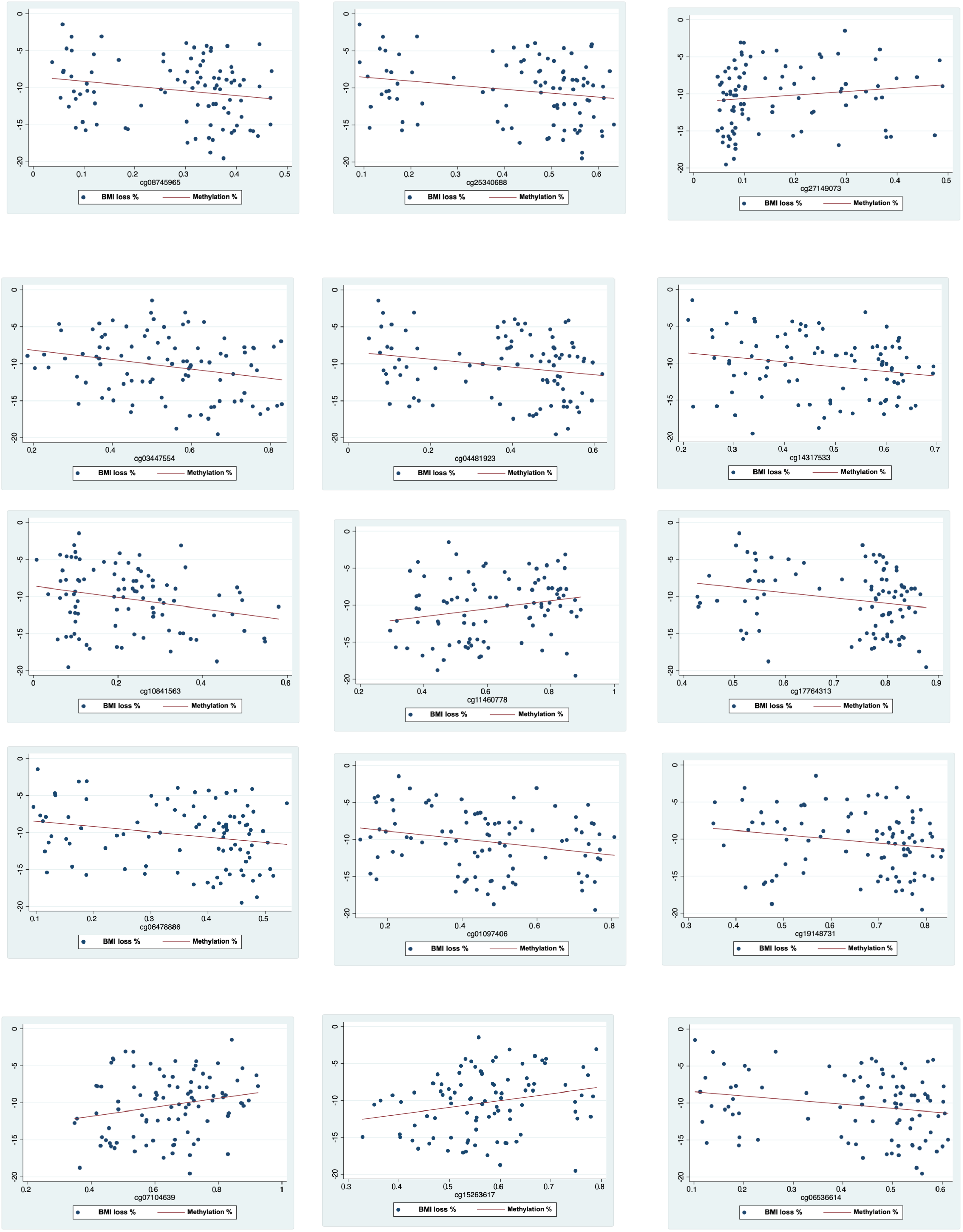
cg08745965
BMI loss %
Methylation %
cg25340688
BMI loss %
Methylation %
cg27149073
BMI loss %
Methylation %
cg03447554
BMI loss %
Methylation %
cg04481923
BMI loss %
Methylation %
cg14317533
BMI loss %
Methylation %
cg10841563
BMI loss %
Methylation %
cg11460778
BMI loss %
Methylation %
cg17764313
BMI loss %
Methylation %
cg06478886
BMI loss %
Methylation %
cg01097406
BMI loss %
Methylation %
cg19148731
BMI loss %
Methylation %
cg07104639
BMI loss %
Methylation %
cg15263617
BMI loss %
Methylation %
cg06536614
BMI loss %
Methylation %

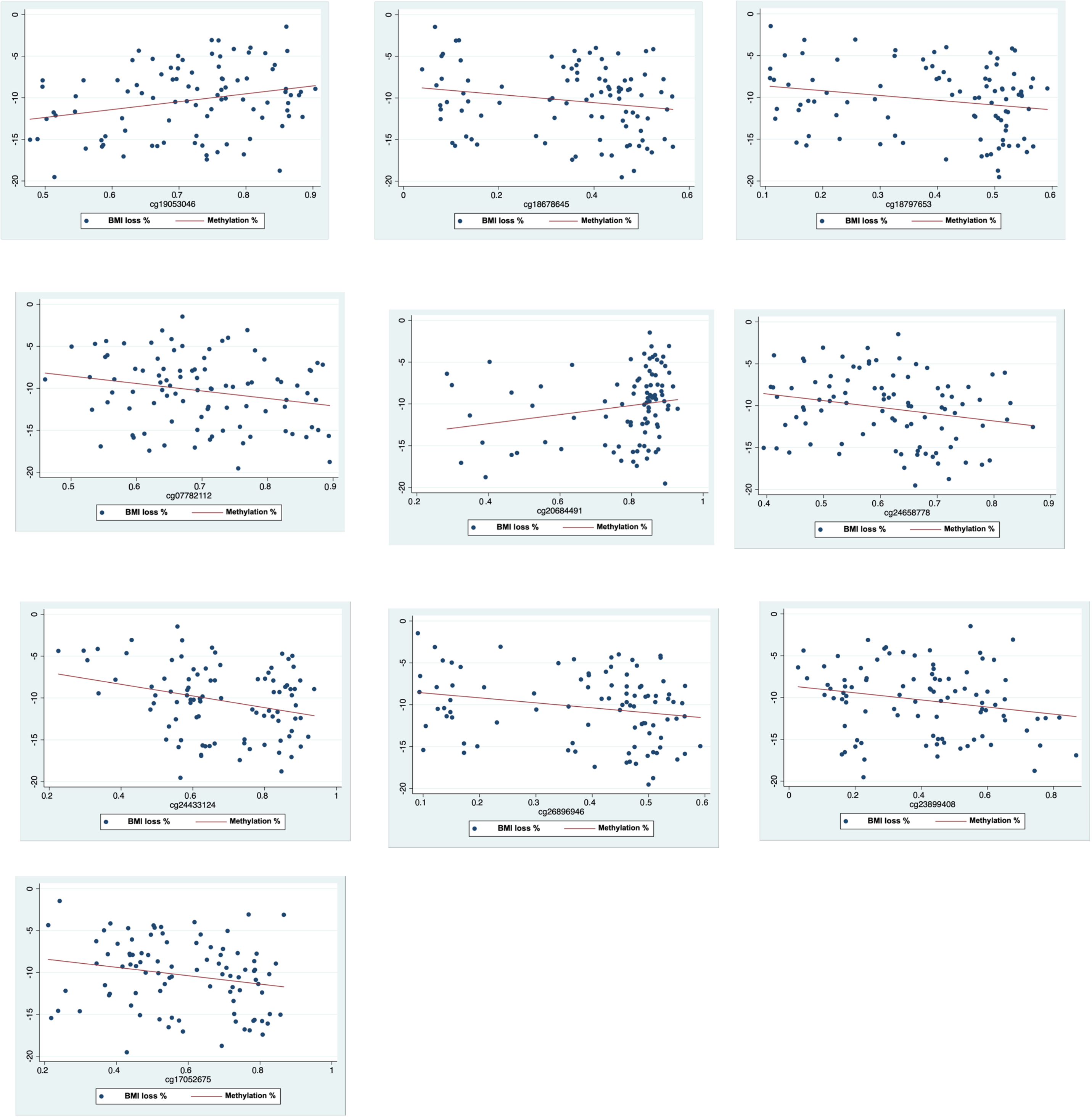
cg19053046
BMI loss %
Methylation %
cg18678645
BMI loss %
Methylation %
cg18797653
BMI loss %
Methylation %
cg07782112
BMI loss %
Methylation %
cg20684491
BMI loss %
Methylation %
cg24658778
BMI loss %
Methylation %
cg24433124
BMI loss %
Methylation %
cg26896946
BMI loss %
Methylation %
cg23899408
BMI loss %
Methylation %
cg17052675
BMI loss %
Methylation %

Supplementary Figures S2. Scatter plots between methylation and change in BMI for each of these LF diet CpG sites.

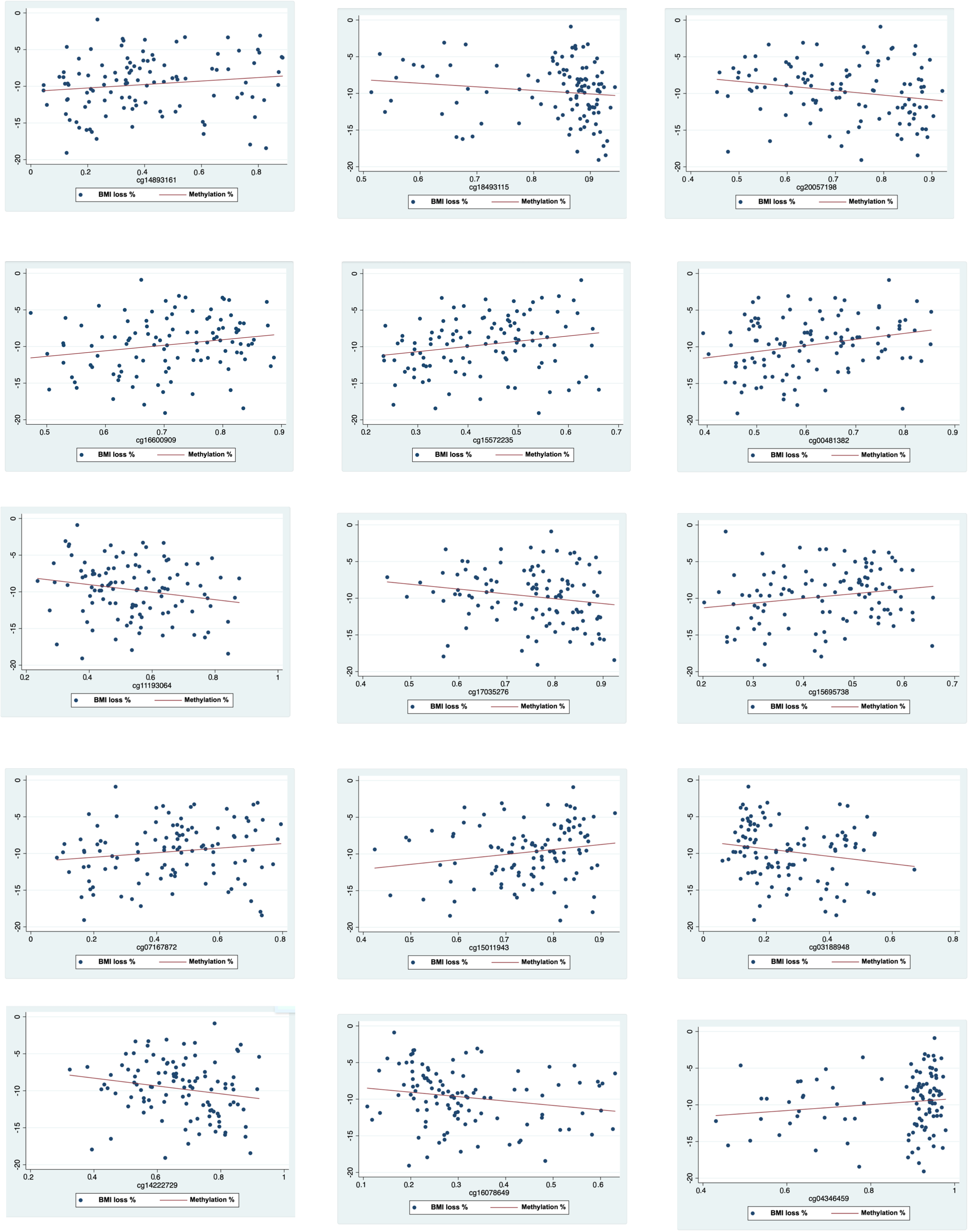

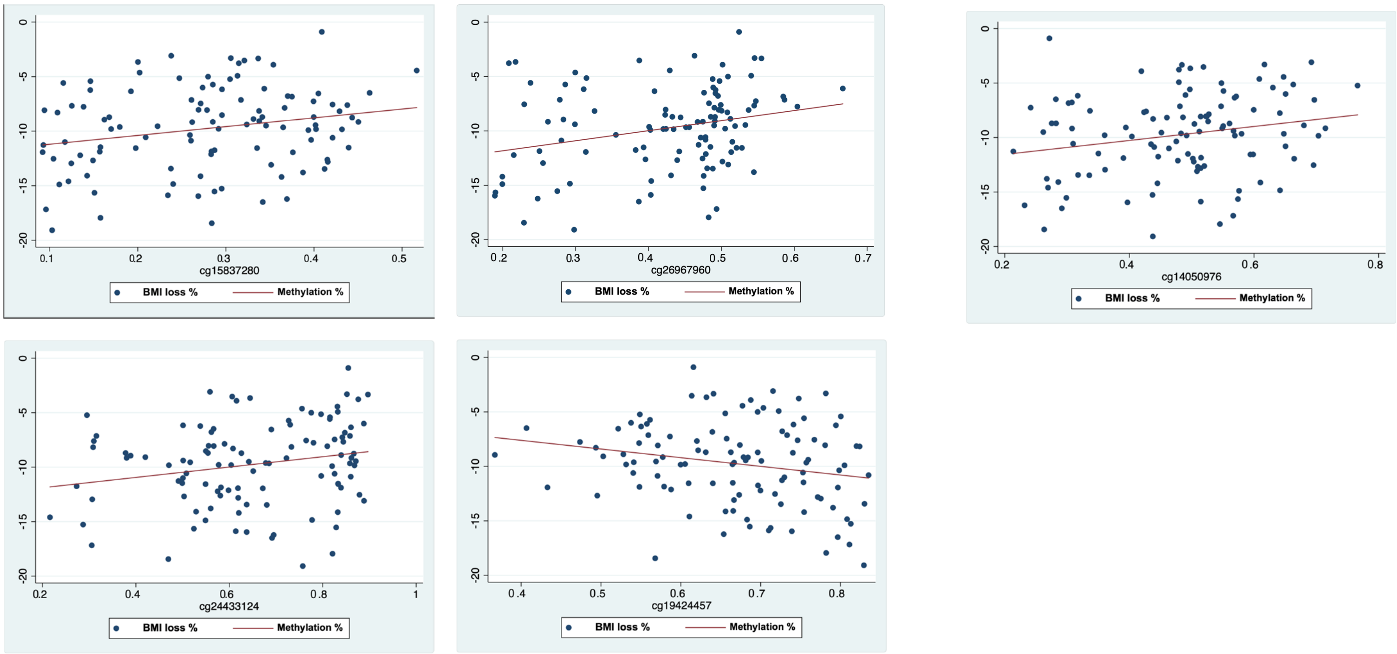
cg15837280
BMI loss %
Methylation %
cg26967960
BMI loss %
Methylation %
cg14050976
BMI loss %
Methylation %
cg24433124
BMI loss %
Methylation %
cg19424457
BMI loss %
Methylation %
